# Prevalence, Pathogenicity, Virulence, Antibiotic Resistance, and Phylogenetic Analysis of Biofilm-Producing *Listeria monocytogenes* Isolated from Different Ecological Niches in Egypt: Food, Humans, Animals, and Environment

**DOI:** 10.3390/pathogens9010005

**Published:** 2019-12-18

**Authors:** Kamelia M. Osman, Anthony D. Kappell, Edward M. Fox, Ahmed Orabi, Ahmed Samir

**Affiliations:** 1 Department of Microbiology, Faculty of Veterinary Medicine, Cairo University, Cairo 12211, Egypt; drorabi2012@yahoo.com (A.O.); ahmedsamir121@hotmail.com (A.S.); 2 Water Quality Center, Department of Civil, Construction and Environmental Engineering, Marquette University, Milwaukee, WI 53233, USA; adkappell@gmail.com; 3 Department of Applied Sciences, North Umbria University, Newcastle upon Tyne NE1 2SU, UK; Edward.fox@northumbria.ac.uk

**Keywords:** *L. monocytogenes*, humans, animals, food, antimicrobial and virulence genes, bioinformatics analysis, *prfA* phylogenetic analysis

## Abstract

Serious outbreaks of foodborne disease have been caused by *Listeria monocytogenes* found in retail delicatessens and the severity of disease is significant, with high hospitalization and mortality rates. Little is understood about the formidable public health threat of *L. monocytogenes* in all four niches, humans, animals, food, and environment, in Egypt. This study analyzed the presence of *L. monocytogenes* collected from the four environmental niches and bioinformatics analysis was implemented to analyze and compare the data. PCR was used to detect virulence genes encoded by pathogenicity island (LIPI-1). *prf*A amino acid substation that causes constitutive expression of virulence was common in 77.7% of isolates. BLAST analysis did not match other isolates in the NCBI database, suggesting this may be a characteristic of the region associated with these isolates. A second group included the NH1 isolate originating in China, and BLAST analysis showed this *prf*A allele was shared with isolates from other global locations, such as Europe and North America. Identification of possible links and transmission pathways between the four niches helps to decrease the risk of disease in humans, to take more specific control measures in the context of disease prevention, to limit economic losses associated with food recalls, and highlights the need for treatment options.

## 1. Introduction

*Listeria monocytogenes* is a facultative intracellular pathogen, which may be classified as asapronoses (or saprozoonoses) with its environmental reservoir [1], where it may grow effectively outside of the host and to infect a relatively wide range of hosts (polyhostality) [1]. *L. monocytogenes* is present in natural ecosystems and is widely disseminated in different environmental niches [2]. It has been isolated from humans, and more than 50 species of wild and domestic animals, including mammals, birds, fish, crustaceans, and ticks, in addition to environmental sources, such as animal silage, soil, plants, sewage, stream water, and processing environments [3,4,5,6]. Although the genus *Listeria* includes many species, due to the pathogenicity of *L. monocytogenes* in human hosts and its ability to flourish in hostile environments, earlier research implicating genome sequencing were to a great extent concentrated on *L. monocytogenes* [7,8,9,10,11]. *L. monocytogenes* is implicated with human listeriosis and in the present decennium, numerous serious outbreaks of foodborne listeriosis have been recorded in different countries and continents [11,12,13,14,15,16], causing serious manifestations in the form of septicemia and meningitis, principally in the immunocompromised and old populaces in addition to pregnant women, who may bring forth stillborn babies or seriously contaminated infants [4,9,17,18,19,20,21,22,23,24,25,26].

The variance between *L. monocytogenes* strains in their virulence potentiality, that is consistent with the flagellar antigen groups, was exhibited in vitro and in vivo. Minimally, three distinct genetic lineages, and identified alphanumerically, were distinctive [27,28]: lineage I: which comprises serotypes causing pandemic listeriosis, lineage II: which embraces serotypes responsible for occasional listeriosis, and lineage III: which includes serotypes isolated from incidents of listeriosis on rare occasions. When the *Listeria* that were isolated from human, animal, and food were serotyped, 95% of the isolates were identified to be 1/2a, 1/2b and 4b [20,27,28,29]. The expression of several crucial virulence factors [8] are implicated in the pathogenesis of *L. monocytogenes*, which is harmonized and controlled by the regulatory *prf*A gene [8,30]. It has been hypothetically proposed that the shift of *Listeria* species from a facultative pathogen to saprobe [7] was due to losing its virulence genes, of which the *prf*A cluster is taken as an example, during natural selection of the *Listeria* species, giving the impression to believe that *Listeria* inclines to develop by preferably losing its virulence more readily than its possession of virulence traits.

Analysis of *L. monocytogenes* isolated from the farm milieu and its farm animals showed that some *L. monocytogenes*, which are correlated with human infection, circulate within the biosphere and the agroecosystems, contributing to the propagation of these pathogens throughout the food chain [9], thus posing a major health issue [9]. Ready-to-eat (RTE) foods, which include processed foods, are of special interest, as they are exposed to the processing milieu prior to packaging and molded for human consumption in the absence of any control measures capable of inactivating the bacterium, such as cooking or being stored at refrigerated or chilled temperatures, which allows growth of *L. monocytogenes* to high numbers. *L. monocytogenes* prevalence in the food milieu may not really be relative to its frequency in the food commodities [9,31].

In order to improve understanding of the pathogen’s ecology, the present endeavor was aimed to investigate *L. monocytogenes* contingency in human and animal clinical cases, retail food, and environment to establish their potential virulence and antibiotic resistance in Egypt. Consequently, this might permit to distinguish potential connections and conveyance routes amid these niches, the diversity in virulence, resistance, and additional features of clinical significance, allowing *L. monocytogenes* to become an intimidating epidemiological hazard and consequently, facilitate the prevention of humans and animals infection, to implement a specified and detailed course of action in the state of affairs in preventive medicine and to minimize financial losses accompanying food recalls.

## 2. Results

The current study examined the frequency of *L. monocytogenes* in different retail food and in animals or human clinical cases of abortion and septicemia in the Greater Cairo Area, based on the prevalence as determined in previous national studies on milk samples [32,33,34]. *L. monocytogenes* were found in 20/1607 (1.3%) samples analyzed (including 1406 retail food samples, 136 veterinaries, and 65 clinical samples). The results recorded in Table 1, and Supplementary Tables S1 and S2 disclose the frequency of *L. monocytogenes* in the different samples: Milk by-products, kariesh cheese (1/120); Chicken, broilers internal organs (3/120) and layers internal organs (3/120); Table eggs (1/100); Meat, meat by-products (hamburger 1/50); Ducks internal organs (1/60); Silage (3/90); Fish, frozen fish (1/100), fish filet (1/58), herring (1/66); Brain tissue, rabbit (1/30); fetal livers (goats 1/15); Septicemia (ewes 1/24 and women 1/65).

**Figure 1:**
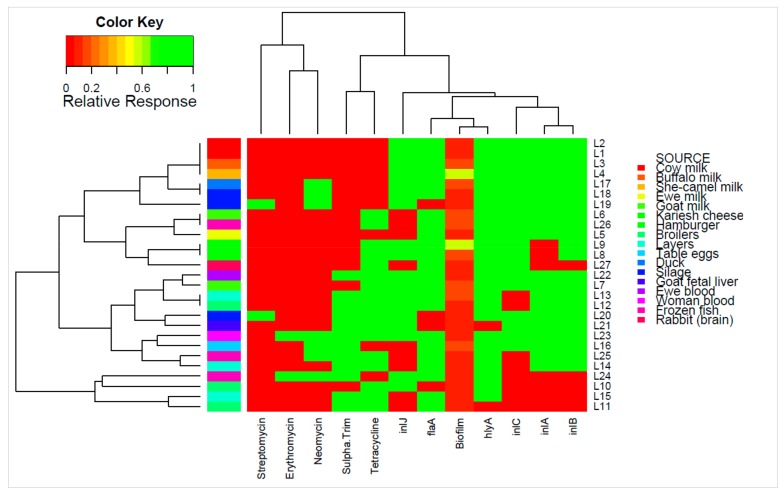
Individual isolates showing hierarchical clustering of isolates and factors. Binary factors (such as antibiotics or genes) indicating presence as green (relative response 1) or absence as red (relative response 0). Clustering is based on Wald-like test (D_2_) and for binary data.

The 20 isolated *L. monocytogenes* along with seven additional isolates from previous surveillance of milk [32,33,34] were examined for differences in serotype, genotypic virulence, and phenotypic virulence, biofilm formation, and antibiotic sensitivity. Table 1 is a condensed version of all results (Supplementary Tables S1 and S2) presenting phenotypic and genotypic differences between the isolates, with Figure 1 presenting a heat map of the isolates to visualize these differences. Nine of the isolates were determined to be serotype 1, while 18 isolates were serotype 4. All 27 isolates demonstrated the presence of genetic elements harbored on the *Listeria* pathogenicity island (LIPI-1; *prfA*, *plcA*, *plcB*, and *actA*), although two isolates demonstrated the absence of listeriolysin O, encoded by the *hlyA* gene (broilers 1/3 and the goat fetal liver). All isolates showed the presence of the minor virulence factor gene, *iap*, encoding the extracellular protein p60. All isolates exhibited causation of keratoconjunctivitis (Anton’s eye test), cytotoxicity of Vero cells, and successful infection and lethality toward mice and chick embryos, indicating that all were virulent isolates of *L. monocytogenes.* The flagellin-encoding *flaA* gene, which contributes to effective invasion during infection [8], was absent in only four of the 27 isolates. PCR screening of four key internalins encoded by *inlA*, *inlB*, *inlC*, and *inlJ* indicated the presence of all four genes in 12 isolates, including the isolates from veterinary and clinical samples, while it was absent in two of the isolates (1/3 from each of the broilers and layers). The frequency of the individual internalin genes *inlA*, *inlB, inlC*, and *inlJ* were 74.1% (20), 81.5% (22), 70.4% (19), and 66.7% (18), respectively. Notably, the isolates (4) from cow, she-camel, and buffalo milk, and the two isolates that caused septicemia, were positive for the presence of all 11 genes examined.

Biofilm formation was determined by Christensen’s test tube method (CT) and microtitre plate assay (MPA). The CT allows for qualitative analysis of attachment to glass surfaces and subsequent biofilm formation, which in our investigation showed moderate to strong biofilm formation by the isolates. The MPA for quantitative analysis of attachment and biofilm formation on plastic surfaces during static conditions displayed strong to very strong biofilm formation ranging from 0.13 to 0.56 of staining, where the reference strains recorded an O.D. of 0.16 (Supplementary Table S1).

The phenotypic antibiotic sensitivity results of the 27 *L. monocytogenes* isolates showed that all were sensitive to all penicillins tested: ampicillin, amoxicillin/clavulanic, amoxicillin, penicillin G, cloxacillin, and oxacillin, the second generation fluroquinolones: ofloxacin, enrofloxacin, and ciprofloxacin, the aminoglycosides: amikacin, gentamicin, kanamycin, and spiramycin, the glucopeptide, vancomycin, and rifamycin. All isolates were resistant to the fluroquinolones: flumequine and perfloxacin, the phenicol: chloramphenicol, the cephalosporins: cefotaxime and cephalothin, the lincosamides: lincomycin and clindamycin, and the polypeptide: bacitracin. The isolates showed 25.9% (7), 7.4% (2), 62.9% (17), 44.4% (12), and 7.4% (2) resistance to neomycin, streptomycin, tetracycline, sulphamethozole-trimethoprim, and erythromycin, respectively. The range of the multiple antibiotic resistance index (MAR*_index_*) was from 0.28 to 0.43, with the greatest MAR*_index_* attributed to the isolate from a case of human septicemia, which showed resistance to 12 antibiotics, including four of the five antibiotics showing variance within the isolates.

**Figure 2:**
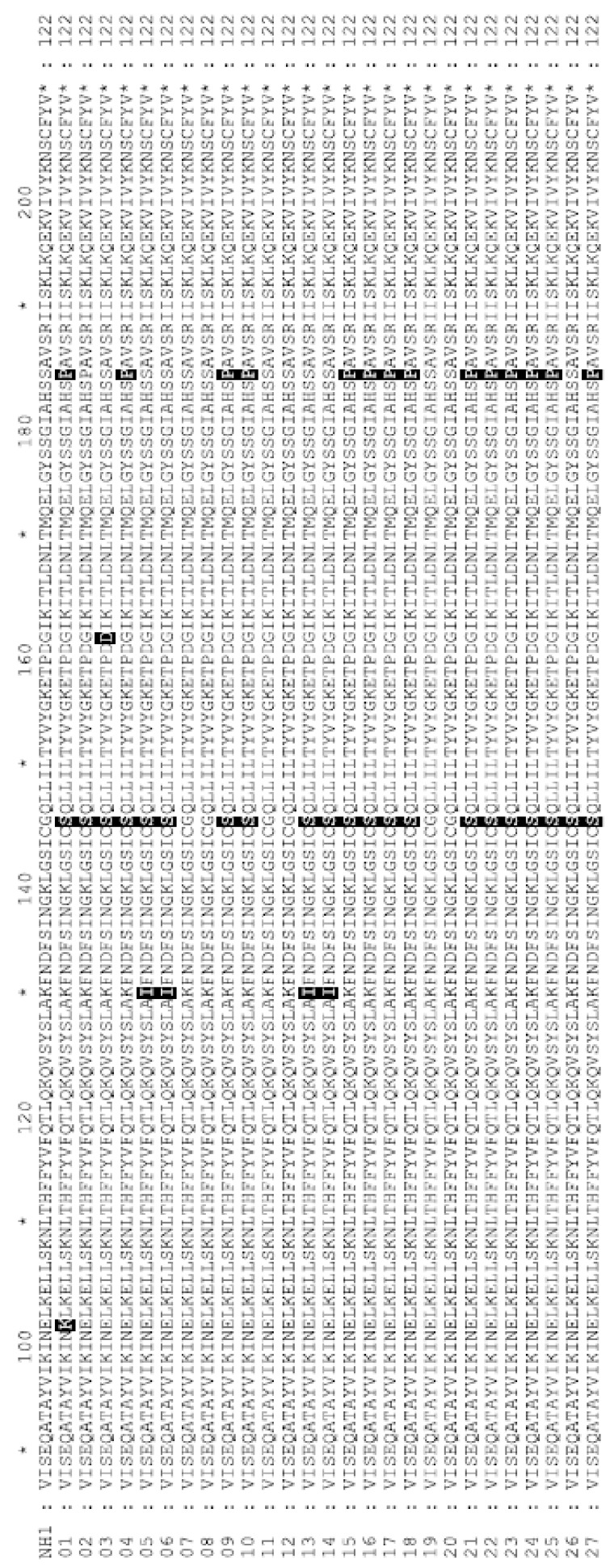
Protein alignment of *prf*A protein from *L. monocytogenes*isolates. Alignment was made to *prf*A from *Listeria monocytogenes*strain NH1 (Genebank: GCA_002969195.1). Differences to NH1 are highlighted and can be found in Table 1.

The *prf*A gene encodes a transcriptional activator of the determinants of pathogenicity in *L. monocytogenes.* The *prf*A gene was sequenced to determine possible differences in virulence and pathogenicity. There were seven nucleotide differences detected between codons 87 and 208 which caused five changes in the amino acid sequences compared to wild-type (Figure 2). Two of the changes were synonymous substitutions in the third position of codons for S127 and T170 in an isolate for cow milk and buffalo milk, respectively. The other five mutations were missense mutations. One isolate from cow milk showed a mutation of E101K and one isolate showed a mutation of G161D. The E101K and G161D mutations were not identified in the non-redundant protein database at NCBI by BLAST nor the literature. Of the 21 isolates demonstrating the mutation of G145S, only one was absent of additional mutations. The G145S mutation was present with K130I in four isolates, in one isolate which also harbored a G161D mutation, and in all 15 isolates with S184P mutations.

**Figure 3:**
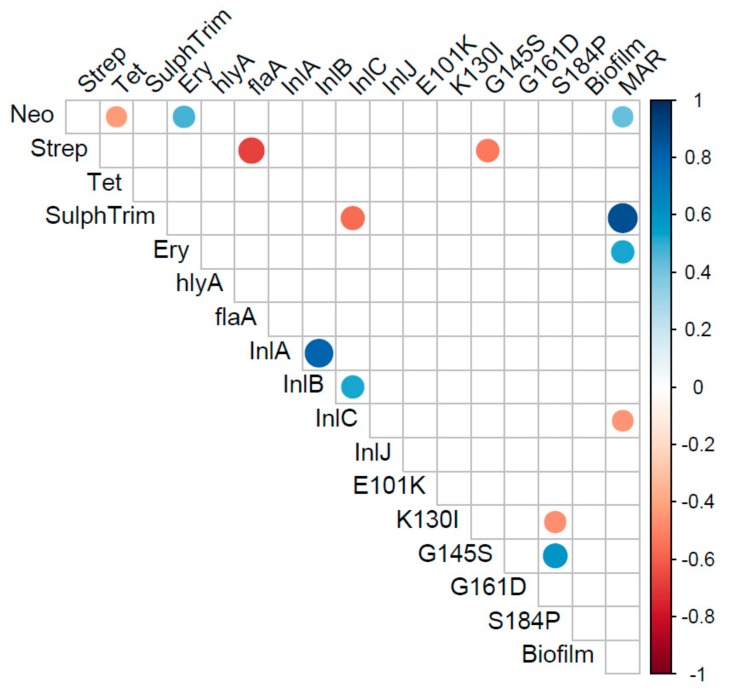
Correlation matrix of virulence and antibiotic resistance profiles that were different among the recovered *Listeria monocytogenes*. Only correlations that were significant (*p* < 0.05) are represented in the matrix.

**Figure 4:**
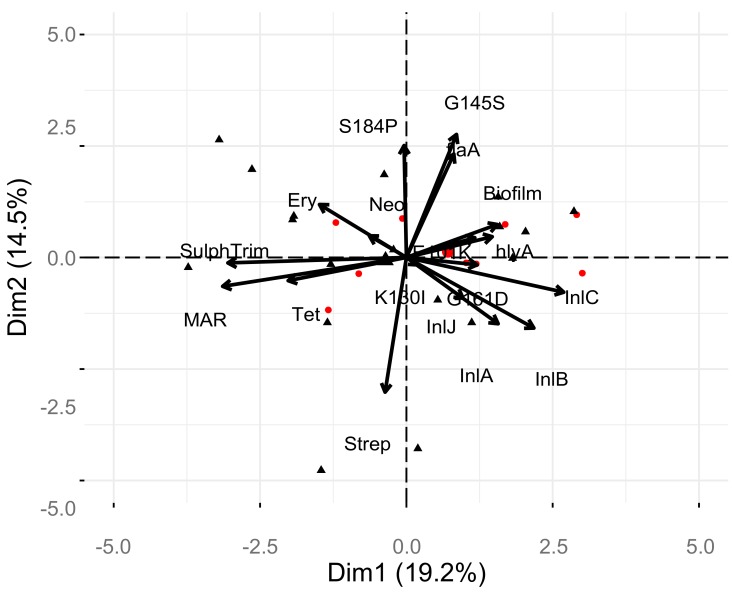
Principle component analysis of factors and relationship with serotype and individual isolates.

Examination of correlations between the phenotypic and genotypic characterizations of the 27 isolates of *L.* monocytogenes was performed (Figure 3). There was a significant positive correlation of the MAR*_index_* with resistance to neomycin, sulphamethozole-trimethoprim, and erythromycin (*p* < 0.05). The strongest correlation of the MAR*_index_* was with the antibiotic combination of sulphamethozole-trimethoprim, suggesting this resistance is most important in multi-antibiotic resistance of *L. monocytogenes.* The presence of the S184P mutation in the *pfr*A gene was positively and strongly correlated with the G145S mutation, as expected based on initial analysis (*p* < 0.05). There was a significant negative correlation of the S184P and K130I mutations in *pfr*A gene (*p* < 0.05). Correlations of genes and phenotypes that are assumed to be independent of one-another included a strong negative correlation of streptomycin resistance with the flagellin encoding, *fla*A, gene and the *pfr*A gene with the G145S mutation (*p* < 0.05). There was also a significant negative correlation between the internalin encoded by the *inl*C gene and the antibiotic combination of sulphamethozole-trimethoprim. PCA analysis (Figure 4) supports a number of these correlations, however, MANOVA analysis indicated no statistical differences in the levels of individual test and there were no strong differences in the test based on source or serotype (*p* > 0.56).

### prfA Phylogenetic Analysis

**Figure 5:**
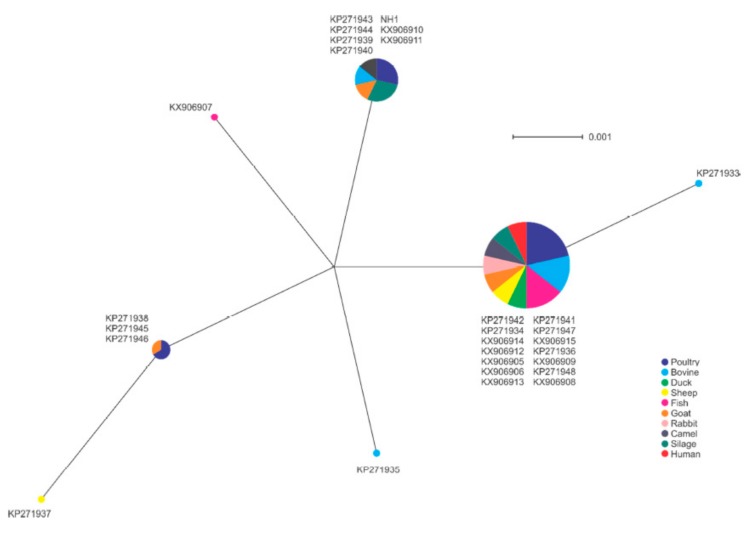
Phylogenetic analysis of *prfA* gene sequences among isolates in this study. Node size indicates proportion of isolates sharing a specific genotype.

The 27 isolates were clustered into seven unique *prf*A alleles. The largest group included 14 isolates, which included isolates of each of the sample types, including the human isolate. BLAST analysis of this allele did not match other isolates in the NCBI database (https://blast.ncbi.nlm.nih.gov/Blast.cgi), suggesting this may be a characteristic of the region associated with these isolates (Figure 5). The second largest group included the NH1 isolate originating in China, and BLAST analysis showed this allele was shared with isolates from other global locations, such as Europe and North America (data not shown). No clear association of a single sample type and specific allele was apparent, with multiple alleles identified among most animal sources included.

## 3. Discussion

The assessment of the actual prevalence of the diverse serotypes causing listeriosis and analyzing the environmental distribution and their bionomics are principal steps to elucidate any potential linkage allying environmental reservoirs of *L. monocytogenes* to human disease. In a review published by Walland et al. [35], the paper outlines listeriosis in animals and exchanges views on/about the scarcity in our knowledge concerning the reservoirs harboring *L. monocytogenes* and its cycling between animal and human hosts. This insufficient understanding is caused by gaps in the available literature on the genetic subtypes and its attribution to the *L. monocytogenes* bionomics, virulence, risk factors, in vivo diagnostics, and listerial pathogeny in animals. The ecology of *L. monocytogenes* serotypes in the reservoir of agricultural and animals’ environments is not known in Egypt.

The general low prevalence of *L. monocytogenes* of less than one percent detected within this study is lower than previous studies, which reflects increased awareness, vigilance, and good practices in food preparation in the Egyptian community in the area of study [3,9,11,36,37,38,39,40,41]. A recent study by Leong et al. [42] of small food businesses in Ireland showed a similar decrease over the course of a three-year study. Leong et al. [42] found a comparable food and environmental occurrence of *L. monocytogenes* at 4.2% and 3.8%, respectively. The highest occurrence was within meat at 7.5% and the lowest in seafood at 1.8%. A similar trend was observed in the current study with occurrence of 2.4% in meat and 1.3% in seafood. The occurrence of *L. monocytogenes* in raw meat, milk, and cheese in this study was lower than that found in other countries [4,11,16,39,40,41,42,43,44,45]. The global *L. monocytogenes* population diversity in the implicated food vehicles could be attributed to several factors: (i) improvements in detection methodologies, (ii) packing facility, (iii) increases in populations of lactic acid bacteria, yeasts, and molds that might have an impact on the microenvironment supporting growth of *L. monocytogenes*, (iv) period and temperature degree of the storage condition, (v) handling by the consumers, (vi) unhygienic conditions, (vii) uncontrolled temperature, (viii) glove/hand issues, (ix) environmental (hygienic conditions of the farms, hygienic conditions of the slaughter houses, rodents, workers, the slicer, trash handling, and cleanup operations) contamination and subsequent cross contamination to other products, (x) transport, (xi) in the processing facilities, and (xii) during handling at the outlets. In the cheese industry, where ripening and storage are critical stages of modernization of the process, several additional parameters must be taken into consideration, including [40]: (i) time needed to ripen, (ii) temperature during storage in the store place, (iii) degree of the primary bacterial load, (iv) post processing contamination, (v) physio-chemical conditions of the cheese process, and (vi) packaging.

Globally, the prevalence rates in fish show great variability [3,25,44,46,47,48,49]: The prevalence of *L. monocytogenes* in fish are presumably from contaminated waters or during manipulation and processing with contaminated environment and/or equipment [47,50]. The *L. monocytogenes* isolated from our frozen fish samples indicates that *L. monocytogenes* has the ability to endure freezing of food, thereby acting as a reservoir to participate in future human listerial outbreaks [9,25,51,52,53].

The presence of the genes of LPI-1, including *prf*A, *plc*A, *plc*B, and *act*A detected in all isolates highlights the potential for virulence by the isolates, which was further confirmed by infection and mortality of mice, chick embryos, and Vero cells [54]. The lack of detection of the listerolysin O encoding *hly*A gene of LIPI-1, required for intracellular pathogenesis, in two of the 27 isolates may suggest a decreased sequence homology, indicating evolutionary plasticity of the *hly*A gene [55]. A similar lack of detection of this gene was observed by Al-Nabulsi et al. [56] and Ndahi et al. [57]. The 27 *L. monocytogenes* isolates exhibited hemolysis on sheep blood agar plates and with the *Staphylococcus aureus* in the CAMP test. Yet, we detected two hemolytic *L. monocytogenes* isolates (recovered from broiler and fetal liver), while at the same time, we were not able to detect the listeriolysin *hyl*A gene in these two isolates, which was previously encountered with serotype 4, as manifested in our results [58], indicating that hemolysis by *L. monocytogenes* could occur by other unrecovered hemolysins. Therefore, en route to explain our recorded phenomenon, Cotter et al. [59] identified a second haemolysin Listeriolysin S (LLS) existing in a subdivision of strains of lineage I, which is the evolutionary lineage of *L. monocytogenes* that assists in the majority of unpremeditated listerial epidemic outbreaks. Thus, the presence of LIPI-1 in all of the isolates indicates a potential health risk with the associated contaminated food products.

Studies of the functions of flagellar-related proteins in *L. monocytogenes* are in the preliminary stage. Although the characteristic tumbling motility of *L. monocytogenes* was observed in all of our 27 isolates, the *fla*A gene was not detected from four of these motile isolates, stipulating that there are alternative mechanisms controlling the flagella and the secretion of other flagella-related proteins involved in the synthesis of the *L. monocytogenes* flagellum required for its motility and the secretion of supplementary flagellar-related proteins [60]. Attachment and formation of biofilm allows the colonization of surfaces, including food processing environments [61]. The presence of LIPI-1 genes, *hly* and *prf*A, is required for efficient biofilm formation by *L. monocytogenes* [62]; however, the current study was unable to detect a correlation between the strength of attachment and biofilm formation and detection of the *hly*A gene or amino acid substitutions in *prf*A. There was also no positive correlation with the absence of the flagellin-encoding *fla*A gene and biofilm formation. The isolates lacking the *fla*A gene did not show an increase in biofilm formation; however, several genes play a role in biofilm formation which were not screened [63]. Likewise, the presence of strong biofilm formation in two isolates might be due to disruption or overexpression of genes with a role in biofilm formation [63]. Further exploration of differences between environmental and clinical isolates in relation to attachment and biofilm formation will lead to greater understanding of the reservoirs of *L. monocytogenes.*

An important element of the LIPI-1 virulence determinant is the *prf*A gene. The *pfr*A gene encodes the key regulator (the PfrA protein) involved in the activation of pathogenicity determinants in *L. monocytogenes*. The high representation of the G145S substitution in *pfr*A suggests that the majority of isolates would have putative constitutive overexpression of virulence factors [64]. However, this overexpression in virulence can lead to decreased fitness outside the host and decrease environmental survival [65]. Additional mutations of K130I, G161D, or S184P were present in PrfA in isolates with the G145S mutation. Substitution of K130 with glutamine (K130Q) caused complete loss of *prf*A activity [66]; however, the isoleucine (K130I) substitution may not have disrupted the putative functional pocket in which it is found, or the G145S substitution causing constitutive activity was not abolished by K130I. The detection of the S184P mutation within 14 of the 21 isolates with the G145S mutation is interesting as the S184 forms direct hydrogen bonds with the nucleotides within the major groove of the DNA binding site [67]. An alanine substitution of S184 (S184A) decreases DNA binding and virulence in mouse model infections [67], however the S184P mutation did not lead to changes in infection of mice or chick embryos in the current study. The G161D mutation was in one isolate and it most likely does not influence the DNA binding domain of *prf*A, however, G161 is conserved with the G180 of *srv*, a PrfA-like Group A *Streptococcus* regulator of virulence, within the putative DNA-binding domain [68]. The detected negative correlations between the G145S mutation and streptomycin resistance was driven by two isolates from silage that were streptomycin-resistant and had no detected mutations within the PrfA protein, suggesting that this was an artifact of low isolate number. The understanding of the benefit and cost of these mutations of PrfA on the regulation of LIPI-1 mediated virulence in the host and environment will inform models of potential risk of environmental strains of *L. monocytogenes.*

In addition to the LIPI-1, the internalin family of proteins are necessary for attachment and host cell invasion in non-phagocytic cells. Internalins are associated with or anchored to the cell wall or may be secreted and together mediate diverse functions in virulence. Currently, only the surface proteins *inl*A and *inl*B are linked with internalization per se [69]. *inl*A is necessary for the recognition and invasion of epithelial cells and is essential in invasion of enterocytes and to crossing the placental barrier, while *inl*B is important for liver and spleen colonization but does not appear necessary for crossing the intestinal epithelium [69]. The small secreted internalin *inl*C may contribute to *inl*A-mediated internalization and *inl*J contributes to virulence after intravenous infections; however, *inl*C and *inl*J are not known to affect internalization, intracellular proliferation, nor cell-to-cell spread [69]. The isolates showed differences in carriage of the internalins encoded by the *inl*A, *inl*B, *inl*C, and *inl*J genes. There were 12 isolates with all four of these internalins, while seven isolates lacked *inl*A but did carry *inl*B. There were no detected differences in the virulence of the isolates based on infection and mortality of mice, chick embryo, and Vero cells, suggesting that the presence of other determinants present could facilitate an invasive pathology, or the isolates contained a sequence variant of *inl*A or *inl*B not detected in the assay. Interestingly, there was a significant negative correlation of the detection of *inl*C and phenotypic resistance to the antibiotic combination of sulphamethozole-trimethoprim, suggesting that strains with *inl*C may currently have decreased incidences of mechanisms yielding resistance to suplfamethozole-trimethoprim. However, a greater number of isolates and identification of the specific mechanisms of antibiotic resistance will be needed to further support this potential correlation. The absence of genes encoding the *inl*C or *inl*J in only four of the isolates suggests a *L. monocytogenes* population with potentially diverse markers associated with infectivity. *L. monocytogenes* isolates from imported frozen fish lacked the four main internalin genes (*inl*A, *inl*B, *inl*C, and *inl*J) in contrast to the previous studies, where internalin genes were present in almost all previously examined *L. monocytogenes* isolates from all 4 niches—humans, animals, food, and environment [46,47,48,70,71,72,73,74,75,76,77,78].

*L. monocytogenes* exhibiting resistance to antibiotics commonly used for the treatment of listeriosis, including ampicillin and other penicillins, and gentamicin resulting in treatment failure, are of major concern [79]. While antibiotic resistance of *L. monocytogenes* isolates from food, the environment, and human have been reported during the present decade [49], differences between the phenotypic antibiotic resistance of isolates from different ecological niches appear to vary by antimicrobial usage in humans and animals of different geographical location [3,46,50,80]. The isolates in this study showed sensitivity to antibiotics within the penicillin class, second generation fluoroquinolones, glucopeptide class, and rifampicin, while showing complete resistance to phenicols, polypeptides, and lincosamides. The isolates also showed the characteristic intrinsic resistance to cephalosporins and first generation fluoroquinolones [81]. The isolates were variable in their resistance to the macrolide, erythromycin, the aminoglycosides, neomycin and streptomycin, tetracycline, and the combination folic acid pathway inhibitors, sulphamethozole-trimethoprim, indicating acquired resistance from mutation or horizontal gene transfer. The prevalence of 44% (12/27) of isolates showing sulphamethozole-trimethoprim resistance is concerning as there are very few reports of this resistance. Importantly, this is a treatment option utilized in cases of penicillin resistance and so presents a significant risk for human treatment of listeriosis. Bertsch et al. [82] detected the presence of the trimethoprim resistance genes *drf*A, *drf*D, and *drf*G within isolates demonstrating resistance to trimethoprim. Horizontal gene transfer of tetracycline resistance has been identified previously in *L. monocytogenes* in relation to the *tet*(M) gene on a Tn*916* family conjugative transposons [82] and plasmid [83] and *tet*(S) bearing conjugative plasmid [84]. Plasmid-mediated resistance to erythromycin and streptomycin has also been documented in *L. monocytogenes* [84,85]. The MAR*_index_* indicates the relative level of resistance to antibiotics. The MAR*_index_* was driven by the antibiotic resistance, which was variable in the isolates. The highest MAR*_index_* was identified in the isolate from clinical septicemia with the variable resistance to neomycin, tetracycline, erythromycin, and sulphamethozole-trimethoprim. The majority of higher MAR*_index_* were associated with isolates from chickens (layers or broilers), silage, and frozen fish, which was driven by the presence of resistance to tetracycline and sulphamethozole-trimethoprim. *L. monocytogenes* strains resistant to one or more antibiotics have been recovered from environmental, food, and from sporadic cases of human listeriosis [3,27,28,46,49,50,51,80]. Importantly, the common-use drug treatment combination of ampicillin-gentamicin was not disrupted in any of the isolates, indicating high potential success of treatment. However, the high resistance to sulphamethozole-trimethoprim is concerning.

## 4. Materials and Methods

The present investigation was carried out in accordance with recommendations published by the National Institutes of Health (NRC, 2010) in the updated Guide for the Care and Use of Laboratory Animals. The procedures were approved in accordance with the United Kingdom (UK) Animals (scientific procedures) Act of 1986 prior to experimentation by the Cairo University Ethical Committee.

### 4.1. Food and Clinical Samples

Prevalence studies were carried out in the Greater Cairo Area (GCA), which includes the cities in the Cairo, Giza, and in the Qalyubia Governorates, with a total population estimated at 20,500,000 (as of 2012); area: 1709 km^2^; density: 10,400/km^2^ to detect the presence of *L. monocytogenes* in retail food and from animals or human clinical cases showing septicemia and abortion. A total of 1607 samples were collected from different environmental niches and tested for the presence of *L. monocytogenes* following International Organization for Standards 11290-1 (NF EN ISO 11290-1, 1996) during the year 2016: Pasteurized milk (n = 77); Milk by-products, kariesh cheese (n = 120) and yogurt (n = 70); Chicken, chicken filet (n=100), broilers internal organs (n = 120) and layers internal organs (n = 120); Table eggs (n = 100); Meat, retail meat (n = 100) and meat by-products (hamburger n = 50 and basturma n = 50); Duck internal organs (n=60); Silage (n = 90); Frozen seafood, fish (n = 100), fish filet (n = 58), herring (n = 66) and shrimp (n = 50); Brain tissue, cattle (n = 25), sheep (n = 20) and rabbits (n = 30); Abortion, uterine discharge (cows, n = 20 and ewes n = 10) and fetal livers (cows n = 15 and goats n = 15); Septicemia (cows n = 18, buffaloes n = 14, ewes n = 24, goats n = 20 and women n = 65). Samples consisting of 100 g from different retail stores were kept on ice during transportation and analysis was initiated within 4 h.

### 4.2. Isolation, Identification, and Serotyping of L. monocytogenes

Isolation of *Listeria* was performed as in Osman et al. [32,34]. Briefly, subsamples of 25 g or 25 mL were aseptically transferred into sterile stomacher closure bags containing 500 mL of half-strength Fraser enrichment broth with CCFA supplement (pre-enrichment broth) and homogenized for 1 min. Samples in pre-treatment broth were incubated at 30 °C for 48 h. The pre-enrichment culture was diluted 1:100 into 10 mL of full strength Fraser enrichment broth with CCFA supplement (enrichment broth) and incubated at 35 °C for 48 h. A loopful of the enrichment culture was streaked onto PALCAM *Listeria* agar plates and incubated at 37 °C for 24 to 48 h for presumptive isolation and differentiation for *Listeria* species. Colonies were transferred onto tryptic soy yeast extract agar (TSYEA) and incubated at 30 °C for 24 h. Strains were identified using the API *Listeria* system and the Oxoid Microbact *Listeria* 12L system. As previously described [32,34], isolates were examined for morphological and biochemical characteristics by Gram stain, tumbling motility at 20–25 °C, catalase test, Methyl Red (MR), and Voges-Prosakuer tests, hemolysis determined by 5% sheep blood agar, carbohydrate utilization, CAMP test, and phosphatidylinositol-specific phospholipase C (PI-PLC) assay. The enrichment and isolation resulted in 20 identified *L. monocytogenes.* In addition, seven previously isolated *L. monocytogenes* from raw milk samples collected from ewes, goats, buffaloes, and cows were included in this study [32,34]. Serotype was assayed following the manufacturer’s instructions of commercially available antisera against serovars 1 and 4 (Behringwerke AG). 

### 4.3. Antibiogram Profile

The 27 isolated *L. monocytogenes* were tested for their resistance to 28 antibiotics belonging to 11 drug classes by the Kirby–Bauer disk diffusion antibiotic sensitivity testing using the breakpoints for Staphylococci [86] and interpreted according to the CLSI standards [87] or manufacturer’s instructions.

### 4.4. Pathogenicity and Biofilm Formation

The pathogenicity of the 27 *L. monocytogenes* isolates was accessed, as previously implemented by Osman et al. [32,34], by: Anton’s eye test for purulent keratoconjunctivitis, the infection and mortality of experimental mice and chick embryos, cytotoxicity of Vero cells, and biofilm formation by Christensen’s tube method and microtitre plate assays.

### 4.5. Molecular Confirmation of L. monocytogenes and Detection of Virulence Genes

The 27 isolates, 20 isolates from recent sampling, and the 7 resuscitated from frozen glycerol stocks (40% glycerol stored at −70 °C), freshly grown on TSYEA were used for DNA extraction. DNA extraction and PCR confirmation of species were performed as previously described [33]. Colonies of isolates were boiled in 400 µL of TE (10 mMTris-HCl, 1 mM EDTA, pH 8.0) for 10 min and centrifuged at 14,000 rpm for 10 min. The supernatant was immediately used for PCR reactions. The genus was confirmed by PCR as previously described utilizing primers (Supplementary Table S3) specific for the 16S rRNA gene of *Listeria.* PCR reaction were performed in 50 µL containing 2 µL of DNA extract, 1.5 mM MgCl_2_, 250 µM dNTPs, 1x Ampli PCR buffer, 0.5 µM of each primer, and 1.25 U of AmpliTaq DNA polymerase, and filled with molecular grade water to volume. The reaction was overlaid with mineral oil and tubes were placed in a thermal cycler (Perkin-Elmer Cetus, Norwalk, Conn.). The conditions for amplification were initially denatured for 94 °C for 4 min, followed by 25 cycles of 1 min at 94 °C, 60 °C for 1 min, and 72 °C for 1 min, ending with a final extension for 5 min at 72 °C. *L. monocytogenes* strain (ATCC 19115) and an *E. coli* strain (ATCC 25922) were included as positive and negative controls, respectively. Intragenic regions of 11 virulence genes (*prfA, hlyA, inlA, inlB, inlC, inlJ, plcA, plcB, Iap, actA*, and *flaA*) were examined by PCR. The primers are included in Supplementary Table S3. The *L. monocytogenes* virulence genes form the LIPI-1 pathogenicity island (*plcA, plcB, prfA*, and *actA*), the genes encoding internalin proteins (*inlA, inlB, inlC* and *inlJ*), an adhesion protein (*lap)*, and a flagellin protein (*flaA*). Bacterial isolates and reference strains were subjected to PCR assay. The PCR conditions were optimized to 1 mM of each primer, 0.65 UTaq DNA Polymerase, 0.2 mM dNTPs, 1X PCR buffer, 1.5 mM MgCl_2_, and 2 µL of template in 25 μl final volume. Amplification was performed in a thermal cycler with an initial denaturation step of 95 °C for 5 min, followed by 35 amplification cycles of 15 sec at 95 °C (denaturation), 30 sec at 55 °C (annealing), and 90 sec at 72 °C (primer extension), followed by a final extension step of 72 °C for 10 min. The PCR products were determined by separation by electrophoresis in 1.5% agarose and TAE (Tris-acetate EDTA) buffer. Gels were visualized with a UV transilluminator after staining with ethidium bromide.

Partial *prfA* gene PCR products were purified with a GeneJET PCR Purification Kit, sequenced using BigDye Terminator V3.1 Cycle Sequencing kit with forward and reverse primers following the manufacturer’s instructions, and the resulting reactions were detected by an ABI 3730 xl DNA analyzer. The sequences were trimmed at the 5’ and 3’ end to remove quality less than a confidence of 30 and were aligned to full length *prfA* and merged into a single sequence representing the PCR product. The sequences were aligned using Mega7 [88] software and the Muscle alignment [89] with default settings. Single nucleotide changes and the subsequent amino acid change, if applicable, were visualized in Mega7. The gene sequences of *prfA* gene fragments determined in this study were deposited in the GenBank database under accession numbers: KP271933, KP271934, KP271935, KP271936, KP271937, KP271938, KP271939, KP271940, KP271941, KP271942, KP271943, KP271944, KP271945, KP271946, KP271947, KP271948, KX906905, KX906906, KX906907, KX906908, KX906909, KX906910, KX906911, KX906912, KX906913, KX906914, and KX906915.

### 4.6. Statistical Analysis

Analysis was performed only on factors that showed differences. Antibiotic resistance phenotypic profiles, gene presence, and biochemical results were converted into numerical coding. Sensitivity to an antibiotic was represented as 0 and resistance was represented as 1. Presence or absence of a specific gene (e.g., *hlyA*) were represented as 1 and 0, respectively. Statistical analysis was performed using the open statistical program R [90]. Heatmap representations with dendrograms and partitions were plotted using the function ’heatmap.3’ in the ‘GMD’ package [91]. Correlations for variables were calculated using the ‘cor’ function and ‘cor.test’ function to determine significance. Significant correlations were visualized utilizing the ‘corrplot’ function from the ‘corrplot’ R package [92]. False Discovery Rate function was used to correct *p*-values for multiple comparisons [93]. The R packages ‘FactoMineR’ [94] and ‘factoextra’ [95] were used to perform and visualize principal component analysis (PCA). Multivariant statistical analysis was performed using functions in the R package ‘vegan’ [96]. Binomial similarity matrices were calculated for the isolate profiles using ‘vegdist’ function as input for per mutational multivariate analysis of variance (ANOVA) (MANOVA) analyses using the ‘adonis’ function.

### 4.7. Phylogenetic Analysis of the prfA gene

Partial *prfA* gene PCR products were purified with GeneJET PCR Purification Kit, sequenced using BigDye Terminator V3.1 Cycle Sequencing kit with forward and reverse primers following manufacturer’s instructions, and the resulting reactions were detected by ABI 3730 xl DNA analyzer. The sequences were trimmed at the 5’ and 3’ end to remove quality less than a confidence of 30 and were aligned to full length *prfA* and merged into a single sequence representing the PCR product. The sequences were aligned using Mega7 [88] software and the Muscle alignment [89] with default settings. Single nucleotide changes and the subsequent amino acid change, if applicable, were visualized in Mega7.

The *prf*A nucleotide sequence of each isolate was trimmed, in frame, to 360 bp in length. A translation alignment was performed using Geneious software [97], followed by phylogenetic Neighbor Net analysis using SplitsTree software [98,99].

## 5. Conclusions

While additional isolates would be preferable in a study such as this, the low incidence of isolation is an indication of good practices in food preparation that can be further improved. Additional isolates may have strengthened the detected significant correlations while also identifying additional correlations. To upgrade our perception of the pathogen’s ecology, it will be empirical to execute additional coherent experiments and high-resolution population genomic analysis of *L. monocytogenes* isolated from the four niches—humans, animals, food, and environment. This will allow us to trace the evolutionary origins and to prognosticate if there have been any *L. monocytogenes* host switches which are considered to be a serious hazard to food security. The importance of such studies allows us to speculate the fact that animals could be reservoirs and hotbeds for the blooming of novel virulent *L. monocytogenes* infecting humans, facilitating the assessment of their potential public health significance in addition to the implementation of control steps to prevent diseases to mitigate infection of humans and animals, and to minimize financial deficit accompanying food recalls and animal welfare. Moreover, genetic identification related to the host-pathogen associations, being central to host adaptation, prompts the pharmaceutical companies to advance new medications to control listeriosis.

## Figures and Tables

**Table 1:** Virulence and antibiotic resistance profiles that showed variability among the *L. monocytogenes* isolates in this study.

	Virulence Genotype						Biofilm Formation		*Prf*A Mutations					
Source of Isolated *L. monocytogenes*	*hlyA*	*flaA*	*inlA*	*inlB*	*inlC*	*inlJ*	CT	MPA (O.D.)	E101K	K130I	G145S	G161D	S184P	Accession Numbers
Cow milk	+	+	+	+	+	+	Strong	0.12	+	-	+	-	+	KP271933
Cow milk	+	+	+	+	+	+	Strong	0.12	-	-	+	-	+	KP271934
Buffalo milk	+	+	+	+	+	+	Moderate	0.15	-	-	+	+	-	KP271935
She-camel milk	+	+	+	+	+	+	Strong	0.56	-	-	+	-	+	KP271936
Ewe milk	+	+	+	+	+	ND	Strong	0.11	-	+	+	-	-	KP271937
Goat milk	+	+	+	+	+	ND	Moderate	0.2	-	+	+	-	-	KP271938
Goat milk	+	+	+	+	+	+	Strong	0.2	-	-	-	-	-	KP271939
Kariesh cheese	+	+	ND	+	+	+	Strong	0.16	-	-	-	-	-	KP271940
Hamburger	+	+	ND	+	+	+	Strong	0.56	-	-	+	-	+	KP271941
Broilers	+	ND	ND	ND	ND	+	Strong	0.12	-	-	+	-	+	KP271942
Broilers	ND	+	ND	ND	ND	ND	Strong	0.11	-	-	-	-	-	KP271943
Broilers	+	+	+	+	ND	+	Strong	0.11	-	-	-	-	-	KP271944
Layers	+	+	+	+	ND	+	Moderate	0.2	-	+	+	-	-	KP271945
Layers	+	+	+	+	ND	ND	Strong	0.1	-	+	+	-	-	KP271946
Layers	+	+	ND	ND	ND	ND	Strong	0.12	-	-	+	-	+	KP271947
Table eggs	+	+	+	+	+	ND	Moderate	0.15	-	-	+	-	+	KP271948
Duck	+	+	+	+	+	+	Moderate	0.15	-	-	+	-	+	KX906914
Silage	+	+	+	+	+	+	Strong	0.1	-	-	+	-	+	KX906909
Silage	+	ND	+	+	+	+	Strong	0.1	-	-	-	-	-	KX906910
Silage	+	ND	+	+	+	+	Strong	0.1	-	-	-	-	-	KX906911
Goat fetal liver	ND	ND	+	+	+	+	Strong	0.12	-	-	+	-	+	KX906913
Ewe blood (Septicemia)	+	+	+	+	+	+	Strong	0.12	-	-	+	-	+	KX906912
Woman blood (Septicemia)	+	+	+	+	+	+	Strong	0.12	-	-	+	-	+	KX906908
Frozen fish	+	+	ND	ND	ND	+	Strong	0.1	-	-	+	-	+	KX906905
Frozen fish	+	+	+	+	ND	ND	Strong	0.12	-	-	+	-	+	KX906906
Herring	+	+	+	+	+	ND	Moderate	0.15	-	-	+	-	-	KX906907
Rabbit (brain)	+	+	ND	ND	+	ND	Strong	0.1	-	-	+	-	+	KX906915

ND represents not detected.CT represents Christensen’s tube method.MPA represents microtitre plate assays.O.D. represents optical density.

## Supplementary Materials

The following are available online at https://www.mdpi.com/2076-0817/9/1/5/s1, Table S1: athogenicity profiles of *Listeria monocytogenes* recovered from different samples, Table S1: cont. Pathogenicity profiles of *Listeria monocytogenes* recovered from different samples, Table S2: Antibiogram for *L. monocytogenes* isolates, Table S3: PCR Primers and references for 16S rRNA of *Listeria* and virulence genes. Data S1: Fasta formatted alignments of sequences obtained from MEGA7.
