# Supplementary material for: Prevalence, Pathogenicity, Virulence, Antibiotic Resistance, and Phylogenetic Analysis of Biofilm-Producing Listeria monocytogenes Isolated from Different Ecological Niches in Egypt: Food, Humans, Animals, and Environment
Source: Pathogens. 2019 Dec 18;9(1):5. doi: 10.3390/pathogens9010005 (PMC7168649; doi:10.3390/pathogens9010005)
Supplement: Supplementary file 1 [file pathogens-09-00005-s001.pdf]

Supplemental: Prevalence, pathogenicity, virulence, antibiotic resistance and phylogenetic analysis of biofilm producing *Listeria monocytogenes* isolated from different ecological niches in Egypt: Food, humans, animals and environment

Table S1. Pathogenicity profiles of *Listeria monocytogenes* recovered from different samples

Source of samples	n=. of recovered isolates	Serotype	Pathogenicity profile																		
			CAMP (+/-) with S/R	PI-PLC	Anton's eye test	Mice lethality	Chick-embryo lethality	Vero cell ingestion assay	Biofilm		Virulence genes										
									CT	MPA (O.D.)	***prfA***	***hlyA***	***PLcA***	***pLcB***	***Iap***	***actA***	*fla*A	***Inl*A**	***Inl*B**	***Inl*C**	***Inlj***
**Reference strain (ATCC 7494)**		**4**	**+/-**	**+**	**+**	**+**	**+**	**++**	Strong	Strong (0.16)	+	+	+	+	+	+	+	+	+	+	+
**She-camel milk**	**1**	**4**	**+/-**	**+**	**+**	**+**	**+**	**++**	Strong	Very Strong (0.56)	+	+	+	+	+	+	+	+	+	+	+
**Buffalo milk**	**1**	**1**	**+/-**	**+**	**+**	**+**	**+**	**++**	Moderate	Strong (0.15)	+	+	+	+	+	+	+	+	+	+	+
**Cow milk**	**2**	**1**	**+/-**	**+**	**+**	**+**	**+**	**++**	Strong	Strong (0.13)	+	+	+	+	+	+	+	+	+	+	+
		**4**	**+/-**	**+**	**+**	**+**	**+**	**++**	Strong	Strong (0.14)	+	+	+	+	+	+	+	+	+	+	+
**Goat milk**	**2**	**1**	**+/-**	**+**	**+**	**+**	**+**	**++**	Moderate	Very Strong (0.21)	+	+	+	+	+	+	+	+	+	+	ND
		**4**	**+/-**	**+**	**+**	**+**	**+**	**++**	Strong	Strong (0.14)	+	+	+	+	+	+	+	+	+	+	+
**Ewe milk**	**1**	**4**	**+/-**	**+**	**+**	**+**	**+**	**++**	Strong	Strong (0.12)	+	+	+	+	+	+	+	+	+	+	ND
**Silage**	**3**	**4**	**+/-**	**+**	**+**	**+**	**+**	**++**	Strong	Strong (0.14)	+	+	+	+	+	+	+	+	+	+	+
		**4**	**+/-**	**+**	**+**	**+**	**+**	**++**	Strong	Strong (0.14)	+	+	+	+	+	+	ND	+	+	+	+
		**4**	**+/-**	**+**	**+**	**+**	**+**	**++**	Strong	Strong (0.14)	+	+	+	+	+	+	ND	+	+	+	+
**Rabbits brain**	**1**	**4**	**+/-**	**+**	**+**	**+**	**+**	**++**	Strong	Strong (0.14)	+	+	+	+	+	+	+	ND	ND	+	ND

CAMP: Christie, Atkins, Munch-Petersen test; S/R: *Staphylococcus aureus*/*Rhodococcusequi*; PI-PLC: phosphatidylinositol-specific phospholipase C; O.D.: Optical Density; O.D. $_{595}$<0.1= Weak; O.D. $_{595}$ ≤0.1= Strong; O.D. $_{595}$>1= Very Strong; CT, Christensen's tube; MPA, Microtiter plate assay; ND, not detected

Table S1 cont . Pathogenicity profiles of *Listeria monocytogenes* recovered from different samples

Source of samples	n= of recovered isolates	Serotype	Pathogenicity profile																		
			CAMP (+/-) with S/R	PI-PLC	Anton's eye test	Mice lethality	Chick-embryo lethality	Vero cell ingestion assay	Biofilm		Virulence genes										
									CT	MPA (O.D.)	*prfA*	*hlyA*	*PLcA*	*pLcB*	*Iap*	*actA*	*fla*A	*Inl*A	*Inl*B	*Inl*C	*Inlj*
Kariesh cheese	1	4	+/-	+	+	+	+	++	Strong	Strong (0.16)	+	+	+	+	+	+	+	ND	+	+	+
Hamburger	1	4	+/-	+	+	+	+	++	Strong	Very Strong (0.56)	+	+	+	+	+	+	+	ND	+	+	+
Broilers	3	1	+/-	+	+	+	+	++	Strong	Strong (0.13)	+	+	+	+	+	+	ND	ND	ND	ND	+
		4	+/-	+	+	+	+	++	Strong	Strong (0.14)	+	ND	+	+	+	+	+	ND	ND	ND	ND
		4	+/-	+	+	+	+	++	Strong	Strong (0.14)	+	+	+	+	+	+	+	+	+	ND	+
Layers	3	1	+/-	+	+	+	+	++	Moderate	Very Strong (0.21)	+	+	+	+	+	+	+	+	+	ND	+
		4	+/-	+	+	+	+	++	Strong	Strong (0.14)	+	+	+	+	+	+	+	+	+	ND	ND
		4	+/-	+	+	+	+	++	Strong	Strong (0.17)	+	+	+	+	+	+	+	ND	ND	ND	ND
Table eggs		1	+/-	+	+	+	+	++	Moderate	Strong (0.15)	+	+	+	+	+	+	+	+	+	+	ND
Ducks	1	1	+/-	+	+	+	+	++	Moderate	Strong (0.15)	+	+	+	+	+	+	+	+	+	+	+
Aborted Goat fetal liver	1	1	+/-	+	+	+	+	++	Strong	Strong (0.17)	+	ND	+	+	+	+	ND	+	+	+	+
Septicemic ewe	1	4	+/-	+	+	+	+	++	Strong	Strong (0.17)	+	+	+	+	+	+	+	+	+	+	+
Septicemic women	1	4	+/-	+	+	+	+	++	Strong	Strong (0.17)	+	+	+	+	+	+	+	+	+	+	+
Frozen fish	1	4	+/-	+	+	+	+	++	Strong	Strong (0.14)	+	+	+	+	+	+	+	ND	ND	ND	+
		4	+/-	+	+	+	+	++	Strong	Strong (0.17)	+	+	+	+	+	+	+	+	+	ND	ND
Herring	1	1	+/-	+	+	+	+	++	Moderate	Strong (0.15)	+	+	+	+	+	+	+	+	+	+	ND

CAMP: Christie, Atkins, Munch-Petersen test; S/R: *Staphylococcus aureus*/*Rhodococcusequi*; PI-PLC: phosphatidylinositol-specific phospholipase C; DLABN: DL-alanine-b-naphthylamide HCl; DAPN: D-alanine-p-nitroanilide; O.D.: Optical Density; O.D. $_{595}$<0.1= Weak; O.D. $_{595}$ ≤0.1= Strong; O.D. $_{595}$>1= Very Strong; CT, Christensen's tube; MPA, Microtiter plate assay; ND, not detected

Table S2. Antibiogram for *L. monocytogenes* isolates

n=	Source	Ampicillin (25 µg)	Amoxicillin/clavulanic (10 µg)	Amoxicillin (25 µg)	Penicillin G (10 IU)	Cloxacillin (5 µg)	Oxacillin (1 µg)	Ofloxacin (10 µg)	Enrofloxacin (10 µg)	Ciprofloxacin (5 µg)	Flumequine (30 µg)	Pefloxacin (30 µg)	Amikacin (30 µg)	Gentamicin (10 µg)	Kanamycin (30 µg)	Neomycin (10 µg)	Streptomycin (10 µg)	Chloramphenicol (30 µg)	Tetracycline (30 µg)	Sulpha-trimethoprime 1:19 (25 µg)	Cefotaxime (30 µg)	Cephalothin (30 µg)	Lincomycin (2 µg)	Clindamycin (2 µg)	Bacitracin (10 units)	Vancomycin (30 µg)	Erythromycin (15 µg)	Spiramycin (100 µg)	Rifamycin (5 µg)	MAR_{index}
1	Cow milk	S	S	S	S	S	S	S	S	S	R	R	S	S	S	S	S	R	S	S	R	R	R	R	R	S	S	S	S	0.3
2	Cow milk	S	S	S	S	S	S	S	S	S	R	R	S	S	S	S	S	R	S	S	R	R	R	R	R	S	S	S	S	0.3
3	Buffalo milk	S	S	S	S	S	S	S	S	S	R	R	S	S	S	S	S	R	S	S	R	R	R	R	R	S	S	S	S	0.3
4	She-camel milk	S	S	S	S	S	S	S	S	S	R	R	S	S	S	S	S	R	S	S	R	R	R	R	R	S	S	S	S	0.3
5	Ewe milk	S	S	S	S	S	S	S	S	S	R	R	S	S	S	S	S	R	S	S	R	R	R	R	R	S	S	S	S	0.3
6	Goat milk	S	S	S	S	S	S	S	S	S	R	R	S	S	S	S	S	R	R	S	R	R	R	R	R	S	S	S	S	0.3
7	Goat milk	S	S	S	S	S	S	S	S	S	R	R	S	S	S	S	S	R	R	S	R	R	R	R	R	S	S	S	S	0.3
8	Kariesh cheese	S	S	S	S	S	S	S	S	S	R	R	S	S	S	S	S	R	R	S	R	R	R	R	R	S	S	S	S	0.3
9	Hamburger	S	S	S	S	S	S	S	S	S	R	R	S	S	S	S	S	R	R	S	R	R	R	R	R	S	S	S	S	0.3
10	Broilers	S	S	S	S	S	S	S	S	S	R	R	S	S	S	S	S	R	R	S	R	R	R	R	R	S	S	S	S	0.3
11	Broilers	S	S	S	S	S	S	S	S	S	R	R	S	S	S	S	S	R	R	R	R	R	R	R	R	S	S	S	S	0.4
12	Broilers	S	S	S	S	S	S	S	S	S	R	R	S	S	S	S	S	R	R	R	R	R	R	R	R	S	S	S	S	0.4
13	Layers	S	S	S	S	S	S	S	S	S	R	R	S	S	S	S	S	R	R	R	R	R	R	R	R	S	S	S	S	0.4
14	Layers	S	S	S	S	S	S	S	S	S	R	R	S	S	S	S	S	R	R	R	R	R	R	R	R	S	S	S	S	0.4
15	Layers	S	S	S	S	S	S	S	S	S	R	R	S	S	S	S	S	R	R	R	R	R	R	R	R	S	S	S	S	0.4
16	Table eggs	S	S	S	S	S	S	S	S	S	R	R	S	S	S	R	S	R	S	R	R	R	R	R	R	S	S	S	S	0.4
17	Duck spleen	S	S	S	S	S	S	S	S	S	R	R	S	S	S	R	S	R	S	S	R	R	R	R	R	S	S	S	S	0.3
18	Silage	S	S	S	S	S	S	S	S	S	R	R	S	S	S	R	S	R	S	S	R	R	R	R	R	S	S	S	S	0.3
19	Silage	S	S	S	S	S	S	S	S	S	R	R	S	S	S	R	R	R	S	S	R	R	R	R	R	S	S	S	S	0.4
20	Silage	S	S	S	S	S	S	S	S	S	R	R	S	S	S	S	R	R	R	R	R	R	R	R	R	S	S	S	S	0.4
21	Goat fetal liver	S	S	S	S	S	S	S	S	S	R	R	S	S	S	S	S	R	R	R	R	R	R	R	R	S	S	S	S	0.4
22	Sheep blood (Septicemia)	S	S	S	S	S	S	S	S	S	R	R	S	S	S	S	S	R	R	R	R	R	R	R	R	S	S	S	S	0.4
23	Human blood (Septicemia)	S	S	S	S	S	S	S	S	S	R	R	S	S	S	R	S	R	R	R	R	R	R	R	R	S	R	S	S	0.4
24	Frozen fish	S	S	S	S	S	S	S	S	S	R	R	S	S	S	R	S	R	S	R	R	R	R	R	R	S	R	S	S	0.4
25	Frozen fish	S	S	S	S	S	S	S	S	S	R	R	S	S	S	R	S	R	R	R	R	R	R	R	R	S	S	S	S	0.4
26	Herring	S	S	S	S	S	S	S	S	S	R	R	S	S	S	S	S	R	R	S	R	R	R	R	R	S	S	S	S	0.3
27	Rabbit brain tissue	S	S	S	S	S	S	S	S	S	R	R	S	S	S	S	S	R	R	S	R	R	R	R	R	S	S	S	S	0.3

Table S3: PCR Primers and references for 16S rRNA of *Listeria* and virulence genes

Name	Amplicon Size (bp)	Primer Sequence	References
16S rRNA	938	F: 5’-CAG-CMG-CCG-CGG-TAA-TWC-3’<br>R: 5’-CTC-CAT-AAA-GGT-GAC-CCT-3’	Vazquez-Boland et al. [1]
*prf*A	571	F: 5'-CCCCAAGTAGCAGGACATGCTAA-3'<br>R: 5'-GTATCACAAAGCTCACGAG-3'	Cooray et al. [2]
*hly*A	702	F: 5′-CCTAAGACGCCAATCGAA-3′<br>R: 5′-AAGCGCTTGCAACTGCTC-3′	Mengaud et al. [3]
*inl*A	800	F:5’-ATTATGCCAAGTGGTTACGGA-3’<br>R: 5’-ATCTGTTTGCGAGACCGTGTC-3’	Liu et al. [4]
*inl*B	500	F: 5’CATGGGAGAGTAACCCAACC-3’<br>R: 5’-GCGGTAACCCCTTTGTCATA-3’	Zhang and Knabel [5]
*Inl*C	400	F: 5'-CCCACAATCAAATAAGTGACCTT-3’<br>R: 5'-CTGGGTCTTTGACAGTATTTGTT-3’	
*Inl*J	238	F: 5'-TGTAACCCCGCTTACACAGTT-3’<br>R: 5'-AGCGGCTTGGCAGTCTAATA-3’	Liu et al. [4]
*plc*A	1484	F:5′-CTGCTTGAGCGTTCATGTCTCATCCCCC-3′<br>R: 5′-ATGGGTTTCACTCTCCTTCTAC-3′	Notermans et al. [6]
*plc*B	795	F: 5'-GCAAGTGTTCTAGTCTTTCCGG-3’<br>R: 5'-ACCTGCCAAAGTTTGCTGTGA-3’	Cooray et al. [2]
*act*A	839	F: 5′-CGCCGCGGAAATTAAAAAAAGA-3′<br>R: 5′-ACGAAGGAACCGGGCTGCTAG-3’	Suarez et al. [7]
Iap	131	F:5'-ACAAGCTGCACCTGTTGCAG-3’<br>R: 5'-TGACAGCGTGTGTAGTAGCA-3’	Furrer et al. [8]
*fla*A	420	F: 5'-AGCTCTTAGCTCCATGAGTT-3’<br>R: 5'-AGTAGCAGCACCTGTAGCAGT-3’	Gray and Kroll [9]

Data S1: Fasta formatted alignments of sequences obtained from MEGA7

```
>NH1
ATGAACGCTCAAGCAGAAGAATTCAAAAAATATTTAGAAACTAACGGGATAAAACCAAAACAATTTCATAAAAAAGAACTTATT
TTTAACCAATGGGATCCACAAGAATATTGTATTTTTCTATATGATGGTATCACAAAGCTCACGAGTATTAGCGAGAACGGGACCAT
CATGAATTTACAATACTACAAAGGGGCTTTCGTTATAATGTCTGGCTTTATTGATACAGAAACATCGGTTGGCTATTATAATTTAG
AAGTCATTAGCGAGCAGGCTACCGCATACGTTATCAAAATAAACGAACTAAAAGAACTACTGAGCAAAAATCTTACGCACTTTTT
CTATGTTTTCCAAACCCTACAAAAACAAGTTTCATACAGTCTAGCTAAATTTAATGATTTTTCGATTAACGGGAAGCTTGGCTCTA
TTTGCGGTCAACTTTTAATCCTGACCTATGTGTATGGTAAAGAAACTCCTGATGGCATCAAGATTACACTGGATAATTTAACAATG
CAGGAGTTAGGATATTCAAGTGGCATCGCACATAGCTCAGCTGTTAGCAGAATTATTTCCAAATTAAAGCAAGAGAAAGTTATCG
TGTATAAAAATTCATGCTTTTATGTACAAAATCTTGATTATCTCAAAAGATATGCCCCTAAATTAGATGAATGGTTTTATTTAGCAT
GTCCTGCTACTTGGGGAAAATTAAATTAA-------------------------------------------------------------------------------------------------------------------
>Cow_Milk_KP271933
-------------------------------------------------------------------------------------------------------------------------------------------------------------------------------------
----------------------------------------------------------------------------------------
ATTAGCGAGCAGGCTACCGCATACGTTATCAAAATAAACAAACTAAAAGAACTACTGAGCAAAAATCTTACGCACTTTTTCTATG
TTTTCCAAACCCTACAAAAACAAGTTTCATACAGTCTAGCTAAATTTAATGATTTTTCGATTAACGGGAAGCTTGGCTCTATTTGC
AGTCAACTTTTAATCCTGACCTATGTGTATGGTAAAGAAACTCCTGATGGCATCAAGATTACACTGGATAATTTAACAATGCAGGA
GTTAGGATATTCAAGTGGCATCGCACATAGCCCAGCTGTTAGCAGAATTATTTCCAAATTAAAGCAAGAGAAAGTTATCGTGTAT
AAAAATTCATGCTTTTATGT-------------------------------------------------------------------------------------------------------------------------------------------
----------------------------------------------------------------------
>Cow_Milk_KP271934
----------------------------------------------------------------------------------------------------------------------------------------------------
CACGAGTATTAGCGAGAACGGGACCATCATGAATTTACAATACTACAAAGGGGCTTTCGTTATAATGTCTGGCTTTATTGATACAG
AAACATCGGTTGGCTATTATAATTTAGAAGTCATTAGCGAGCAGGCTACCGCATACGTTATCAAAATAAACGAACTAAAAGAACT
ACTGAGCAAAAATCTTACGCACTTTTTCTATGTTTTCCAAACCCTACAAAAACAAGTTTCATACAGTCTAGCTAAATTTAATGATTT
TTCGATTAACGGGAAGCTTGGCTCTATTTGCAGTCAACTTTTAATCCTGACCTATGTGTATGGTAAAGAAACTCCTGATGGCATCA
AGATTACACTGGATAATTTAACAATGCAGGAGTTAGGATATTCAAGTGGCATCGCACATAGCCCAGCTGTTAGCAGAATTATTTC
CAAATTAAAGCAAGAGAAAGTTATCGTGTATAAAAATTCATGCTTTTATGTACAAAATCTTGATTATCTCAAAAGATATGCCCCTA
AATTAGATGAATGGTTTTATTTA-------------------------------------------------------------------------------------------------------------------------------------
----------------
>Buffallo_Milk_KP271935
-----------------------------------------------------------------------------------------------------------------------------------------------------
ACGAGTATTAGCGAGAACGGGACCATCATGAATTTACAATACTACAAAGGGGCTTTCGTTATAATGTCTGGCTTTATTGATACAG
AAACATCGGTTGGCTATTATAATTTAGAAGTCATTAGCGAGCAGGCTACCGCATACGTTATCAAAATAAACGAACTAAAAGAACT
ACTGAGCAAAAATCTTACGCACTTTTTCTATGTTTTCCAAACCCTACAAAAACAAGTTTCATACAGTCTAGCTAAATTTAATGATTT
TTCGATTAACGGGAAGCTTGGCTCTATTTGCAGTCAACTTTTAATCCTGACCTATGTGTATGGTAAAGAAACTCCTGATGACATCA
AGATTACACTGGATAATTTAACAATGCAGGAGTTAGGATATTCAAGTGGCATCGCACATAGCTCAGCTGTTAGCAGAATTATTTCC
AAATTAAAGCAAGAGAAAGTTATCGTGTATAAAAATTCATGCTTTTATGTACAAAATCTTGATTATCTCAAAAGATATGCCCCTAA
ATTAGATGAATGGTTTTATTTAG-------------------------------------------------------------------------------------------------------------------------------------
---------------
>She-camel_Milk_KP271936
------------------------------------------------------------------------------------------------------------------------------------------------------------------
AGAACGGGACCATCATGAATTTACAATACTACAAAGGGGCTTTCGTTATAATGTCTGGCTTTATTGATACAGAAACATCGGTTGGC
TATTATAATTTAGAAGTCATTAGCGAGCAGGCTACCGCATACGTTATCAAAATAAACGAACTAAAAGAACTACTGAGCAAAAATC
TTACGCACTTTTTCTATGTTTTCCAAACCCTACAAAAACAAGTTTCATACAGTCTAGCTAAATTTAATGATTTTTCGATTAACGGGA
AGCTTGGCTCTATTTGCAGTCAACTTTTAATCCTGACCTATGTGTATGGTAAAGAAACTCCTGATGGCATCAAGATTACACTGGAT
AATTTAACAATGCAGGAGTTAGGATATTCAAGTGGCATCGCACATAGCCCAGCTGTTAGCAGAATTATTTCCAAATTAAAGCAAG
AGAAAGTTATCGTGTATAAAAATTCATGCTTTTATGTACAAAATCTTGATTATCTCAAAAGATATGCCCCTAAATTAGATGAATGG
TTTTATTTAGCATGT------------------------------------------------------------------------------------------------------------------------------------------------
>Ewe_Milk-KP271937
-------------------------------------------------------------------------------------------------------------------------------------------------------
GAGTATTAGCGAGAACGGGACCATCATGAATTTACAATACTACAAAGGGGCTTTCGTTATAATGTCTGGCTTTATTGATACAGAA
ACATCGGTTGGCTATTATAATTTAGAAGTCATTAGCGAGCAGGCTACCGCATACGTTATCAAAATAAACGAACTAAAAGAACTAC
TGAGCAAAAATCTTACGCACTTTTTCTATGTTTTCCAAACCCTACAAAAACAAGTTTCATACAGTCTAGCTATATTTAATGATTTTT
CGATTAACGGGAAGCTTGGCTCTATTTGCAGTCAACTTTTAATCCTGACCTATGTGTATGGTAAAGAAACTCCTGATGGCATCAAG
ATTACACTGGATAATTTAACGATGCAGGAGTTAGGATATTCAAGTGGCATCGCACATAGCTCAGCTGTTAGCAGAATTATTTCCAA
ATTAAAGCAAGAGAAAGTTATCGTGTATAAAAATTCATGCTTTTATGTACAAAATCTTGATTATCTCAAAAGATATGCCCCTAAAT
TAGATGAATGGTTTTATTTAG-----------------------------------------------------------------------------------------------------------------------------------------
-----------
>Goat_Milk_KP271938
-------------------------------------------------------------------------------------------------------------------------------------------------------
GAGTATTAGCGAGAACGGGACCATCATGAATTTACAATACTACAAAGGGGCTTTCGTTATAATGTCTGGCTTTATTGATACAGAA
ACATCGGTTGGCTATTATAATTTAGAAGTCATTAGCGAGCAGGCTACCGCATACGTTATCAAAATAAACGAACTAAAAGAACTAC
TGAGCAAAAATCTTACGCACTTTTTCTATGTTTTCCAAACCCTACAAAAACAAGTTTCATACAGTCTAGCTATATTTAATGATTTTT
CGATTAACGGGAAGCTTGGCTCTATTTGCAGTCAACTTTTAATCCTGACCTATGTGTATGGTAAAGAAACTCCTGATGGCATCAAG
ATTACACTGGATAATTTAACAATGCAGGAGTTAGGATATTCAAGTGGCATCGCACATAGCTCAGCTGTTAGCAGAATTATTTCCAA
ATTAAAGCAAGAGAAAGTTATCGTGTATAAAAATTCATGCTTTTATGTACAAAATCTTGATTATCTCAAAAGATATGCCCCTAAAT
TAGATGAATGGTTTTATTTAGCATG---------------------------------------------------------------------------------------------------------------------------------
---------------
```

>Goat_Milk_KP271939

CACGAGTATTAGCGAGAACGGGACCATCATGAATTTACAATACTACAAAGGGGCTTTCGTTATAATGTCTGGCTTTATTGATACAG
AAACATCGGTTGGCTATTATAATTTAGAAGTCATTAGCGAGCAGGCTACCGCATACGTTATCAAAATAAACGAACTAAAAGAACT
ACTGAGCAAAAATCTTACGCACTTTTTCTATGTTTTCCAAACCCTACAAAAACAAGTTTCATACAGTCTAGCTAAATTTAATGATTT
TTCGATTAACGGGAAGCTTGGCTCTATTTGCGGTCAACTTTTAATCCTGACCTATGTGTATGGTAAAGAAACTCCTGATGGCATCA
AGATTACACTGGATAATTTAACAATGCAGGAGTTAGGATATTCAAGTGGCATCGCACATAGCTCAGCTGTTAGCAGAATTATTTCC
AAATTAAAGCAAGAGAAAGTTATCGTGTATAAAAATTCATGCTTTTATGTACAAAATCTTGATTATCTCAAAAGATATGCCCCTAA
ATTAGATGAATGGTTTTATTTA--

>Kariesch_cheese_KP271940

TATTAGCGAGAACGGGACCATCATGAATTTACAATACTACAAAGGGGCTTTCGTTATAATGTCTGGCTTTATTGATACAGAAACAT
CGGTTGGCTATTATAATTTAGAAGTCATTAGCGAGCAGGCTACCGCATACGTTATCAAAATAAACGAACTAAAAGAACTACTGAG
CAAAAATCTTACGCACTTTTTCTATGTTTTCCAAACCCTACAAAAACAAGTTTCATACAGTCTAGCTAAATTTAATGATTTTTCGAT
TAACGGGAAGCTTGGCTCTATTTGCGGTCAACTTTTAATCCTGACCTATGTGTATGGTAAAGAAACTCCTGATGGCATCAAGATTA
CACTGGATAATTTAACAATGCAGGAGTTAGGATATTCAAGTGGCATCGCACATAGCTCAGCTGTTAGCAGAATTATTTCCAAATTA
AAGCAAGAGAAAGTTATCGTGTATAAAAATTCATGCTTTTATGTACAAAATCTTGATTATCTCAAAAGATATGCCCCTAAATTAGA
TGAATGGTTTTATTTAGCA--

>Hamburger_KP271941

ACGAGTATTAGCGAGAACGGGACCATCATGAATTTACAATACTACAAAGGGGCTTTCGTTATAATGTCTGGCTTTATTGATACAG
AAACATCGGTTGGCTATTATAATTTAGAAGTCATTAGCGAGCAGGCTACCGCATACGTTATCAAAATAAACGAACTAAAAGAACT
ACTGAGCAAAAATCTTACGCACTTTTTCTATGTTTTCCAAACCCTACAAAAACAAGTTTCATACAGTCTAGCTAAATTTAATGATTT
TTCGATTAACGGGAAGCTTGGCTCTATTTGCAGTCAACTTTTAATCCTGACCTATGTGTATGGTAAAGAAACTCCTGATGGCATCA
AGATTACACTGGATAATTTAACAATGCAGGAGTTAGGATATTCAAGTGGCATCGCACATAGCCCAGCTGTTAGCAGAATTATTTC
CAAATTAAAGCAAGAGAAAGTTATCGTGTATAAAAATTCATGCTTTTATGTACAAAATCTTGATTATCTCAAAAGATATGCCCCTA
AATTAGATGAATGGTTTTATTTAGCAT--

>Broilers_KP271942

CACGAGTATTAGCGAGAACGGGACCATCATGAATTTACAATACTACAAAGGGGCTTTCGTTATAATGTCTGGCTTTATTGATACAG
AAACATCGGTTGGCTATTATAATTTAGAAGTCATTAGCGAGCAGGCTACCGCATACGTTATCAAAATAAACGAACTAAAAGAACT
ACTGAGCAAAAATCTTACGCACTTTTTCTATGTTTTCCAAACCCTACAAAAACAAGTTTCATACAGTCTAGCTAAATTTAATGATTT
TTCGATTAACGGGAAGCTTGGCTCTATTTGCAGTCAACTTTTAATCCTGACCTATGTGTATGGTAAAGAAACTCCTGATGGCATCA
AGATTACACTGGATAATTTAACAATGCAGGAGTTAGGATATTCAAGTGGCATCGCACATAGCCCAGCTGTTAGCAGAATTATTTC
CAAATTAAAGCAAGAGAAAGTTATCGTGTATAAAAATTCATGCTTTTATGTACAAAATCTTGATTATCTCAAAAGATATGCCCCTA
AATTAGATGAATGGTTTTATTTAGCA--

>Broilers_KP271943

CACGAGTATTAGCGAGAACGGGACCATCATGAATTTACAATACTACAAAGGGGCTTTCGTTATAATGTCTGGCTTTATTGATACAG
AAACATCGGTTGGCTATTATAATTTAGAAGTCATTAGCGAGCAGGCTACCGCATACGTTATCAAAATAAACGAACTAAAAGAACT
ACTGAGCAAAAATCTTACGCACTTTTTCTATGTTTTCCAAACCCTACAAAAACAAGTTTCATACAGTCTAGCTAAATTTAATGATTT
TTCGATTAACGGGAAGCTTGGCTCTATTTGCGGTCAACTTTTAATCCTGACCTATGTGTATGGTAAAGAAACTCCTGATGGCATCA
AGATTACACTGGATAATTTAACAATGCAGGAGTTAGGATATTCAAGTGGCATCGCACATAGCTCAGCTGTTAGCAGAATTATTTCC
AAATTAAAGCAAGAGAAAGTTATCGTGTATAAAAATTCATGCTTTTATGTACAAAATCTTGATTATCTCAAAAGATATGCCCCTAA
ATTAGATGAATGGTTTTATTTAGCA--

>Broilers_KP271944

TCACGAGTATTAGCGAGAACGGGACCATCATGAATTTACAATACTACAAAGGGGCTTTCGTTATAATGTCTGGCTTTATTGATACA
GAAACATCGGTTGGCTATTATAATTTAGAAGTCATTAGCGAGCAGGCTACCGCATACGTTATCAAAATAAACGAACTAAAAGAAC
TACTGAGCAAAAATCTTACGCACTTTTTCTATGTTTTCCAAACCCTACAAAAACAAGTTTCATACAGTCTAGCTAAATTTAATGATT
TTTCGATTAACGGGAAGCTTGGCTCTATTTGCGGTCAACTTTTAATCCTGACCTATGTGTATGGTAAAGAAACTCCTGATGGCATC
AAGATTACACTGGATAATTTAACAATGCAGGAGTTAGGATATTCAAGTGGCATCGCACATAGCTCAGCTGTTAGCAGAATTATTT
CCAAATTAAAGCAAGAGAAAGTTATCGTGTATAAAAATTCATGCTTTTATGTACAAAATCTTGATTATCTCAAAAGATATGCCCCT
AAATTAGATGAATGGTTTTATTTAGCATG---

>Layers_KP271945

CACGAGTATTAGCGAGAACGGGACCATCATGAATTTACAATACTACAAAGGGGCTTTCGTTATAATGTCTGGCTTTATTGATACAG
AAACATCGGTTGGCTATTATAATTTAGAAGTCATTAGCGAGCAGGCTACCGCATACGTTATCAAAATAAACGAACTAAAAGAACT
ACTGAGCAAAAATCTTACGCACTTTTTCTATGTTTTCCAAACCCTACAAAAACAAGTTTCATACAGTCTAGCTATATTTAATGATTT
TTCGATTAACGGGAAGCTTGGCTCTATTTGCAGTCAACTTTTAATCCTGACCTATGTGTATGGTAAAGAAACTCCTGATGGCATCA
AGATTACACTGGATAATTTAACAATGCAGGAGTTAGGATATTCAAGTGGCATCGCACATAGCTCAGCTGTTAGCAGAATTATTTCC
AAATTAAAGCAAGAGAAAGTTATCGTGTATAAAAATTCATGCTTTTATGTACAAAATCTTGATTATCTCAAAAGATATGCCCCTAA
ATTAGATGAATGGTTTTATTTAGCA--

> Layers_KP271946

AGCGAGAACGGGACCATCATGAATTTACAATACTACAAAGGGGCTTTCGTTATAATGTCTGGCTTTATTGATACAGAAACATCGG
TTGGCTATTATAATTTAGAAGTCATTAGCGAGCAGGCTACCGCATACGTTATCAAAATAAACGAACTAAAAGAACTACTGAGCAA
AAATCTTACGCACTTTTTCTATGTTTTCCAAACCCTACAAAAACAAGTTTCATACAGTCTAGCTATATTTAATGATTTTTCGATTAA
CGGGAAGCTTGGCTCTATTTGCAGTCAACTTTTAATCCTGACCTATGTGTATGGTAAAGAAACTCCTGATGGCATCAAGATTACAC
TGGATAATTTAACAATGCAGGAGTTAGGATATTCAAGTGGCATCGCACATAGCTCAGCTGTTAGCAGAATTATTTCCAAATTAAA
GCAAGAGAAAGTTATCGTGTATAAAAATTCATGCTTTTATGTACAAAATCTTGATTATCTCAAAAGATATGCCCCTAAATTAGATG
AATGGTTTTATTTAGCA---
-
> Layers_KP271947

ACGAGTATTAGCGAGAACGGGACCATCATGAATTTACAATACTACAAAGGGGCTTTCGTTATAATGTCTGGCTTTATTGATACAG
AAACATCGGTTGGCTATTATAATTTAGAAGTCATTAGCGAGCAGGCTACCGCATACGTTATCAAAATAAACGAACTAAAAGAACT
ACTGAGCAAAAATCTTACGCACTTTTTCTATGTTTTCCAAACCCTACAAAAACAAGTTTCATACAGTCTAGCTAAATTTAATGATTT
TTCGATTAACGGGAAGCTTGGCTCTATTTGCAGTCAACTTTTAATCCTGACCTATGTGTATGGTAAAGAAACTCCTGATGGCATCA
AGATTACACTGGATAATTTAACAATGCAGGAGTTAGGATATTCAAGTGGCATCGCACATAGCCCAGCTGTTAGCAGAATTATTTC
CAAATTAAAGCAAGAGAAAGTTATCGTGTATAAAAATTCATGCTTTTATGTACAAAATCTTGATTATCTCAAAAGATATGCCCCTA
AATTAGATGAATGGTTTTATTTAGCATGT--

>Table_eggs_KP271948

GAGTATTAGCGAGAACGGGACCATCATGAATTTACAATACTACAAAGGGGCTTTCGTTATAATGTCTGGCTTTATTGATACAGAA
ACATCGGTTGGCTATTATAATTTAGAAGTCATTAGCGAGCAGGCTACCGCATACGTTATCAAAATAAACGAACTAAAAGAACTAC
TGAGCAAAAATCTTACGCACTTTTTCTATGTTTTCCAAACCCTACAAAAACAAGTTTCATACAGTCTAGCTAAATTTAATGATTTTT
CGATTAACGGGAAGCTTGGCTCTATTTGCAGTCAACTTTTAATCCTGACCTATGTGTATGGTAAAGAAACTCCTGATGGCATCAAG
ATTACACTGGATAATTTAACAATGCAGGAGTTAGGATATTCAAGTGGCATCGCACATAGCCCAGCTGTTAGCAGAATTATTTCCA
AATTAAAGCAAGAGAAAGTTATCGTGTATAAAAATTCATGCTTTTATGTACAAAATCTTGATTATCTCAAAAGATATGCCCCTAAA
TTAGATGAATGGTTTTATTTAGCATG--

>Duck_KX906914

TATTAGCGAGAACGGGACCATCATGAATTTACAATACTACAAAGGGGCTTTCGTTATAATGTCTGGCTTTATTGATACAGAAACAT
CGGTTGGCTATTATAATTTAGAAGTCATTAGCGAGCAGGCTACCGCATACGTTATCAAAATAAACGAACTAAAAGAACTACTGAG
CAAAAATCTTACGCACTTTTTCTATGTTTTCCAAACCCTACAAAAACAAGTTTCATACAGTCTAGCTAAATTTAATGATTTTTCGAT
TAACGGGAAGCTTGGCTCTATTTGCAGTCAACTTTTAATCCTGACCTATGTGTATGGTAAAGAAACTCCTGATGGCATCAAGATTA
CACTGGATAATTTAACAATGCAGGAGTTAGGATATTCAAGTGGCATCGCACATAGCCCAGCTGTTAGCAGAATTATTTCCAAATT
AAAGCAAGAGAAAGTTATCGTGTATAAAAATTCATGCTTTTATGTACAAAATCTTGATTATCTCAAAAGATATGCCCCTAAATTAG
ATGAATGGTTTTATTTAGCA--

>Silage_KX906909

GTATTAGCGAGAACGGGACCATCATGAATTTACAATACTACAAAGGGGCTTTCGTTATAATGTCTGGCTTTATTGATACAGAAAC
ATCGGTTGGCTATTATAATTTAGAAGTCATTAGCGAGCAGGCTACCGCATACGTTATCAAAATAAACGAACTAAAAGAACTACTG
AGCAAAAATCTTACGCACTTTTTCTATGTTTTCCAAACCCTACAAAAACAAGTTTCATACAGTCTAGCTAAATTTAATGATTTTTCG
ATTAACGGGAAGCTTGGCTCTATTTGCAGTCAACTTTTAATCCTGACCTATGTGTATGGTAAAGAAACTCCTGATGGCATCAAGAT
TACACTGGATAATTTAACAATGCAGGAGTTAGGATATTCAAGTGGCATCGCACATAGCCCAGCTGTTAGCAGAATTATTTCCAAA
TTAAAGCAAGAGAAAGTTATCGTGTATAAAAATTCATGCTTTTATGTACAAAATCTTGATTATCTCAAAAGATATGCCCCTAAATT
AGATGAATGGTTTTATTTAGCA--

> Silage_KX906910

ACGAGTATTAGCGAGAACGGGACCATCATGAATTTACAATACTACAAAGGGGCTTTCGTTATAATGTCTGGCTTTATTGATACAG
AAACATCGGTTGGCTATTATAATTTAGAAGTCATTAGCGAGCAGGCTACCGCATACGTTATCAAAATAAACGAACTAAAAGAACT
ACTGAGCAAAAATCTTACGCACTTTTTCTATGTTTTCCAAACCCTACAAAAACAAGTTTCATACAGTCTAGCTAAATTTAATGATTT
TTCGATTAACGGGAAGCTTGGCTCTATTTGCGGTCAACTTTTAATCCTGACCTATGTGTATGGTAAAGAAACTCCTGATGGCATCA
AGATTACACTGGATAATTTAACAATGCAGGAGTTAGGATATTCAAGTGGCATCGCACATAGCTCAGCTGTTAGCAGAATTATTTCC
AAATTAAAGCAAGAGAAAGTTATCGTGTATAAAAATTCATGCTTTTATGTACAAAATCTTGATTATCTCAAAAGATATGCCCCTAA
ATTAGATGAATGGTTTTATTTA---

> Silage_KX906911

GTATTAGCGAGAACGGGACCATCATGAATTTACAATACTACAAAGGGGCTTTCGTTATAATGTCTGGCTTTATTGATACAGAAAC
ATCGGTTGGCTATTATAATTTAGAAGTCATTAGCGAGCAGGCTACCGCATACGTTATCAAAATAAACGAACTAAAAGAACTACTG
AGCAAAAATCTTACGCACTTTTTCTATGTTTTCCAAACCCTACAAAAACAAGTTTCATACAGTCTAGCTAAATTTAATGATTTTTCG
ATTAACGGGAAGCTTGGCTCTATTTGCGGTCAACTTTTAATCCTGACCTATGTGTATGGTAAAGAAACTCCTGATGGCATCAAGAT
TACACTGGATAATTTAACAATGCAGGAGTTAGGATATTCAAGTGGCATCGCACATAGCTCAGCTGTTAGCAGAATTATTTCCAAAT
TAAAGCAAGAGAAAGTTATCGTGTATAAAAATTCATGCTTTTATGTACAAAATCTTGATTATCTCAAAAGATATGCCCCTAAATTA
GATGAATGGTTTTATTTAGCA--

```
>Goat_fetal_liver_KX906913
----------------------------------------------------------------------------------------------------------------------------------------------------------------
CGAGTATTAGCGAGAACGGGACCATCATGAATTTACAATACTACAAAGGGGCTTTCGTTATAATGTCTGGCTTTATTGATACAGA
AACATCGGTTGGCTATTATAATTTAGAAGTCATTAGCGAGCAGGCTACCGCATACGTTATCAAAATAAACGAACTAAAAGAACTA
CTGAGCAAAAATCTTACGCACTTTTTCTATGTTTTCCAAACCCTACAAAAACAAGTTTCATACAGTCTAGCTAAATTTAATGATTTT
TCGATTAACGGGAAGCTTGGCTCTATTTGCAGTCAACTTTTAATCCTGACCTATGTGTATGGTAAAGAAACTCCTGATGGCATCAA
GATTACACTGGATAATTTAACAATGCAGGAGTTAGGATATTCAAGTGGCATCGCACATAGCCCAGCTGTTAGCAGAATTATTTCC
AAATTAAAGCAAGAGAAAGTTATCGTGTATAAAAATTCATGCTTTTATGTACAAAATCTTGATTATCTCAAAAGATATGCCCCTAA
ATTAGATGAATGGTTTTATTTAGCAT-----------------------------------------------------------------------------------------------------------------------------------------
--------------------
>Ewe_blood_KX906912
---------------------------------------------------------------------------------------------------------------------------------------------------------------
CACGAGTATTAGCGAGAACGGGACCATCATGAATTTACAATACTACAAAGGGGCTTTCGTTATAATGTCTGGCTTTATTGATACAG
AAACATCGGTTGGCTATTATAATTTAGAAGTCATTAGCGAGCAGGCTACCGCATACGTTATCAAAATAAACGAACTAAAAGAACT
ACTGAGCAAAAATCTTACGCACTTTTTCTATGTTTTCCAAACCCTACAAAAACAAGTTTCATACAGTCTAGCTAAATTTAATGATTT
TTCGATTAACGGGAAGCTTGGCTCTATTTGCAGTCAACTTTTAATCCTGACCTATGTGTATGGTAAAGAAACTCCTGATGGCATCA
AGATTACACTGGATAATTTAACAATGCAGGAGTTAGGATATTCAAGTGGCATCGCACATAGCCCAGCTGTTAGCAGAATTATTTC
CAAATTAAAGCAAGAGAAAGTTATCGTGTATAAAAATTCATGCTTTTATGTACAAAATCTTGATTATCTCAAAAGATATGCCCCTA
AATTAGATGAATGGTTTTATTTAGCATG------------------------------------------------------------------------------------------------------------------------------------
-----------------------
>Woman_blood_KX906908
----------------------------------------------------------------------------------------------------------------------------------------------------------------
ACGAGTATTAGCGAGAACGGGACCATCATGAATTTACAATACTACAAAGGGGCTTTCGTTATAATGTCTGGCTTTATTGATACAG
AAACATCGGTTGGCTATTATAATTTAGAAGTCATTAGCGAGCAGGCTACCGCATACGTTATCAAAATAAACGAACTAAAAGAACT
ACTGAGCAAAAATCTTACGCACTTTTTCTATGTTTTCCAAACCCTACAAAAACAAGTTTCATACAGTCTAGCTAAATTTAATGATTT
TTCGATTAACGGGAAGCTTGGCTCTATTTGCAGTCAACTTTTAATCCTGACCTATGTGTATGGTAAAGAAACTCCTGATGGCATCA
AGATTACACTGGATAATTTAACAATGCAGGAGTTAGGATATTCAAGTGGCATCGCACATAGCCCAGCTGTTAGCAGAATTATTTC
CAAATTAAAGCAAGAGAAAGTTATCGTGTATAAAAATTCATGCTTTTATGTACAAAATCTTGATTATCTCAAAAGATATGCCCCTA
AATTAGATGAATGGTTTTATTTAGCATGT----------------------------------------------------------------------------------------------------------------------------------
------------------------
>Frozen_Fish_KX906905
-------------------------------------------------------------------------------------------------------------------------------------------------------------------
AGTATTAGCGAGAACGGGACCATCATGAATTTACAATACTACAAAGGGGCTTTCGTTATAATGTCTGGCTTTATTGATACAGAAA
CATCGGTTGGCTATTATAATTTAGAAGTCATTAGCGAGCAGGCTACCGCATACGTTATCAAAATAAACGAACTAAAAGAACTACT
GAGCAAAAATCTTACGCACTTTTTCTATGTTTTCCAAACCCTACAAAAACAAGTTTCATACAGTCTAGCTAAATTTAATGATTTTTC
GATTAACGGGAAGCTTGGCTCTATTTGCAGTCAACTTTTAATCCTGACCTATGTGTATGGTAAAGAAACTCCTGATGGCATCAAGA
TTACACTGGATAATTTAACAATGCAGGAGTTAGGATATTCAAGTGGCATCGCACATAGCCCAGCTGTTAGCAGAATTATTTCCAA
ATTAAAGCAAGAGAAAGTTATCGTGTATAAAAATTCATGCTTTTATGTACAAAATCTTGATTATCTCAAAAGATATGCCCCTAAAT
TAGATGAATGGTTTTATTTAGCA-----------------------------------------------------------------------------------------------------------------------------------------------
--------------
>Frozen_Fish_KX906906
--------------------------------------------------------------------------------------------------------------------------------------------------------------------------------------------------
-----
CAATACTACAAAGGGGCTTTCGTTATAATGTCTGGCTTTATTGATACAGAAACATCGGTTGGCTATTATAATTTAGAAGTCATTAG
CGAGCAGGCTACCGCATACGTTATCAAAATAAACGAACTAAAAGAACTACTGAGCAAAAATCTTACGCACTTTTTCTATGTTTTCC
AAACCCTACAAAAACAAGTTTCATACAGTCTAGCTAAATTTAATGATTTTTCGATTAACGGGAAGCTTGGCTCTATTTGCAGTCAA
CTTTTAATCCTGACCTATGTGTATGGTAAAGAAACTCCTGATGGCATCAAGATTACACTGGATAATTTAACAATGCAGGAGTTAGG
ATATTCAAGTGGCATCGCACATAGCCCAGCTGTTAGCAGAATTATTTCCAAATTAAAGCAAGAGAAAGTTATCGTGTATAAAAAT
TCATGCTTTTATGTACAAAATCTTGATTATCTCAAAAGATATGCCCCTAAATTAGATGAATGGTTTTATTTAGCATGTCC----------------
----------------------------------------------------------------------------------------------------------------------------------------
>Herring_KX906907
----------------------------------------------------------------------------------------------------------------------------------------------------------------
ACGAGTATTAGCGAGAACGGGACCATCATGAATTTACAATACTACAAAGGGGCTTTCGTTATAATGTCTGGCTTTATTGATACAG
AAACATCGGTTGGCTATTATAATTTAGAAGTCATTAGCGAGCAGGCTACCGCATACGTTATCAAAATAAACGAACTAAAAGAACT
ACTGAGCAAAAATCTTACGCACTTTTTCTATGTTTTCCAAACCCTACAAAAACAAGTTTCATACAGCCTAGCTAAATTTAATGATT
TTTCGATTAACGGGAAGCTTGGCTCTATTTGCAGTCAACTTTTAATCCTGACCTATGTGTATGGTAAAGAAACTCCTGATGGCATC
AAGATTACACTGGATAATTTAACAATGCAGGAGTTAGGATATTCAAGTGGCATCGCACATAGCTCAGCTGTTAGCAGAATTATTT
CCAAATTAAAGCAAGAGAAAGTTATCGTGTATAAAAATTCATGCTTTTATGTACAAAATCTTGATTATCTCAAAAGATATGCCCCT
AAATTAGATGAATGGTTTTATTTAGC-----------------------------------------------------------------------------------------------------------------------------------------
----------------------
>Rabbit_brain_KX906915
--------------------------------------------------------------------------------------------------------------------------------------------------------------------------------------------------
---------------------------------------------------------------
CATCGGTTGGCTATTATAATTTAGAAGTCATTAGCGAGCAGGCTACCGCATACGTTATCAAAATAAACGAACTAAAAGAACTACT
GAGCAAAAATCTTACGCACTTTTTCTATGTTTTCCAAACCCTACAAAAACAAGTTTCATACAGTCTAGCTAAATTTAATGATTTTTC
GATTAACGGGAAGCTTGGCTCTATTTGCAGTCAACTTTTAATCCTGACCTATGTGTATGGTAAAGAAACTCCTGATGGCATCAAGA
TTACACTGGATAATTTAACAATGCAGGAGTTAGGATATTCAAGTGGCATCGCACATAGCCCAGCTGTTAGCAGAATTATTTCCAA
ATTAAAGCAAGAGAAAGTTATCGTGTATAAAAATTCATGCTTTTATGTACAAAATCTTGATTATCTCAAAAGATATGCCC--------------
---------------------------------------------------------------------------------------------------------------------------------------------------------------------------------
```

References

1. Vazquez-Boland, J.A.; Kuhn, M.; Berche, P.; Chakraborty, T.; Domínguez-Bernal, G.; Goebel, W.; González-Zorn, B.; Wehland, J.; Kreft, J. *Listeria* pathogenesis and molecular virulence determinants. *Clin. Microbiol. Rev.* **2001** *14*, 584–640.

2. Cooray, K. J.; Nishibori, T.; Xiong, H.; Matsuyama, T.; Fujita, M.; Mitsuyama, M. Detection of multiple virulence-associated genes of *Listeria monocytogenes* by PCR in artificially contaminated milk samples. *Appl. Environ.Microbiol.* **1994** *60,* 3023-3026.

3. Mengaud, J.; Vicente, M.F.; Chenevert, J.; Pereira, J.M.; Geoffroy, C.; Gicquel-Sanzey, B.; Baquero, F.; Perez-Diaz, J.C.; Cossart, P. Expression in *Escherichia coli* and sequence analysis of the listeriolysin 0 determinant of *Listeria monocytogenes. Infect.Immun*. **1988** *56,* 766-772.

4. Liu, D., Lawrence, M.L.; Ainsworth, A.J.; Austin, F.W. A multiplex PCR for species- and virulence-specific determination of *Listeria monocytogenes*. *J.Microbiol.Methods*. **2007** *71,* 133–140.

5. Zhang, W.; Knabel, S.J. Multiplex PCR assay simplifies serotyping and sequence typing of *Listeria monocytogenes* associated with human outbreaks. *J. Food Prot*. **2005** *68,* 1907–1910.

6. Notermans, S.H.W.; Dufrenne, J.; Leimeister-Wachter, M.; Domann, E.; Chakraborty, T. Phosphatidylinositol-specific phospholipase C activity as a marker to distinguish between pathogenic and nonpathogenic *Listeria* species. *Appl. Environ. Microbiol*. **1991** *57,* 2666-2670.

7. Suárez, M.; González-Zorn, B.; Vega, Y.; Chico-Calero, I.; Vázquez-Boland, J.A. A role for ActA in epithelial cell invasion by *Listeria monocytogenes*. *Cell Microbiol*. **2001** *3,* 853-864.

8. Furrer, B.; Candrian, U.; Hoefelein, C.; Luethy, J. Detection and identification of *Listeria monocytogenes* in cooked sausage products and in milk by in vitro amplification of haemolysin gene fragments. *J. Appl.Bacteriol*. **1991** *70,* 372–379.

9. Gray, D.I.; Kroll, R.G. Polymerase chain reaction amplification of the flaA gene for the rapid identification of *Listeria* spp. *Lett. Appl. Microbiol*. **1995** *20,* 65–68.
